# An Anthocyanin-Related Glutathione *S*-Transferase, *MrGST1*, Plays an Essential Role in Fruit Coloration in Chinese Bayberry (*Morella rubra*)

**DOI:** 10.3389/fpls.2022.903333

**Published:** 2022-06-08

**Authors:** Lei Xue, Xiaorong Huang, Zehuang Zhang, Qihua Lin, Qiuzhen Zhong, Yun Zhao, Zhongshan Gao, Changjie Xu

**Affiliations:** ^1^Zhejiang Provincial Key Laboratory of Horticultural Plant Integrative Biology, Zhejiang University, Hangzhou, China; ^2^Fruit Research Institute, Fujian Academy of Agricultural Sciences, Fuzhou, China; ^3^Center of Economic Botany, Core Botanical Gardens, Chinese Academy of Sciences, Wuhan, China

**Keywords:** anthocyanins, Chinese bayberry, transport, glutathione *S*-transferase, MrMYB1.1

## Abstract

Chinese bayberry (*Morella rubra*) is a fruit tree economically important in China and accumulates abundant amounts of anthocyanins in fruit as it ripens. Owing to the fact that all anthocyanin containing fruit tissues in Chinese bayberry are edible and anthocyanins can provide various health benefits in human body, the mechanisms underpinning anthocyanin accumulation in this fruit are worthy of investigation. It has been known that in plants anthocyanins are synthesized in the cytoplasmic surface of the endoplasmic reticulum and subsequently transported into the vacuole for storage, and glutathione *S*-transferases (GSTs) have been verified to be involved in this process. But the characterization and functionalization of the GST counterpart in Chinese bayberry is not available. The GST anthocyanin transporter *MrGST1* was discovered to be related with anthocyanin accumulation in fruit from distinct developmental stages of “Biqi,” a staple cultivar that accumulates over 1 mg/g anthocyanins in ripe fruit. The expression of *MrGST1* was well associated with anthocyanin accumulation either in fruit collected at six developmental stages or in ripe fruit from 12 cultivars. *MrGST1* was found to be responsible for the transport of anthocyanins but not proanthocyanidins when the Arabidopsis *tt19* mutant was functionally complemented. Transient ectopic expression of *MrGST1* in combination with *MrMYB1.1* and *MrbHLH1* dramatically boosted pigmentation in *Nicotiana tabacum* leaves in contrast to *MrMYB1.1* and *MrbHLH1*. The promoter of *MrGST1* comprised eight MYB binding sites (MBSs) according to *cis*-element analysis. Data from yeast one-hybrid assay and dual-luciferase tests demonstrated that MrMYB1.1 exerted considerable transactivation effect on the *MrGST1* promoter by recognizing the MBS4, the fourth MBS from the ATG start site. Our results together provided molecular evidence for the contribution of *MrGST1* in regulating anthocyanin accumulation in Chinese bayberry fruit.

## Introduction

Anthocyanins are water-dissolvable flavonoid pigments detected in a wide variety of plant parts, comprising seeds, roots, leaves, flowers and fruits, and are in charge of a broad range of colors from pink to purple (Mol et al., 1998). They are thought to serve a critical function in the life and development of plants, including attracting pollinating insects and seed-spreading animals, guarding against biotic and abiotic stimuli, absorbing potentially damaging UV radiation, serving as antioxidants, and delaying senescence processes (Chalker-Scott, 1999; Harborne and Williams, 2000; Hoch et al., 2001; Gould, 2004; Hoballah et al., 2007). The anti-inflammatory and antioxidant activities of anthocyanins, which are abundant in numerous edible fruits and vegetables, make them extremely beneficial to human health (Mink et al., 2007). Anthocyanins are generated in most plant species *via* a branch of the flavonoid biosynthesis pathway with a set of catalytic enzymes (Koes et al., 2005; Hichri et al., 2011). These enzymes consist of phenylalanine ammonia lyase (PAL), chalcone synthase (CHS), chalcone isomerase (CHI), flavanone 3-hydroxylase (F3H), Flavonoid 3′-hydroxylase (F3′H), dihydroflavonol 4-reductase (DFR), anthocyanidin synthase (ANS), and UDP-glucose/flavonoid 3-O-glucosyltransferase (UFGT; Winkel-Shirley, 2002; Koes et al., 2005; Tanaka et al., 2008). These enzymes are encoded by structural genes that are regulated by a core regulatory MYB-bHLH-WDR (MBW) complex, which includes MYB transcription factors (TFs), basic helix–loop–helix (bHLH) TFs and WD-repeat proteins (Gonzalez et al., 2008; Jaakola, 2013; Xu et al., 2015). So far, MBW complex that regulates anthocyanin synthesis has been widely reported in plants, including fruit trees such as citrus, apple, pear, peach and kiwifruit (Li et al., 2012; Yao et al., 2017; Huang et al., 2018; Zhou et al., 2019; Rodrigues et al., 2021).

Anthocyanins are transported into the vacuole for storage after being generated on the cytoplasmic surface of the endoplasmic reticulum (ER). Currently, membrane transporters, vesicle trafficking, and glutathione *S*-transferase (GST) mediated transport are the three distinct ways proposed for anthocyanin transport (Zhao and Dixon, 2010; Zhao, 2015). Membrane transporter mainly comprised multidrug and toxic extrusion (MATE) transporters and ATP binding cassette (ABC) protein transporters (Marinova et al., 2007; Francisco et al., 2013). In the case of vesicle trafficking, anthocyanins are sequestered in vesicle-like structures derived from endoplasmic reticulum and later fused into the vacuole *via* cytoskeleton-binding proteins (e.g., GFS9; Poustka et al., 2007; Ichino et al., 2014). GSTs, which are dimeric enzymes catalyzing glutathione (GSH) conjugation to a variety of electrophilic compounds (Dixon et al., 2002b), are of particular interest in the present work. To date, GSTs have been discovered in all known organisms and have been found to be critical in detoxification of xenobiotics, flavonoid metabolism, and biotic and abiotic stress responses (Loyall et al., 2000; Agrawal et al., 2002; Moons, 2005; Pearson, 2005). GSTs are a huge gene family in plants and can be separated into seven subfamilies, namely Tau, dehydro ascorbate reductase (DHAR), tetrachloro hydroquinone dehalogenase (TCHQD), elongation factor 1 gamma (EF1G), Theta, Zeta, and Phi (Dixon et al., 2002b). Except for ZmBz2 in Tau subfamily, other GSTs related to anthocyanin transport that have been reported previously are all clustered in the plant-specific Phi subfamily (Kitamura et al., 2004).

GSTs are perhaps the most essential anthocyanin transporters since their absence results in the phenotype characterized by a noticeable loss of color, such as maize *bz2* (Bronze-2; Marrs et al., 1995), petunia *an9* (Anthocyanin 9; Alfenito et al., 1998), carnation *fl3* (Flavonoid 3; Larsen et al., 2003), and Arabidopsis *tt19* (Transparent Testa 19; Kitamura et al., 2004; Sun et al., 2012). It has been known that TT19 functions as a carrier protein to transport anthocyanins, as well as proanthocyanidins (PA) in seed coat, from the cytosol into the vacuole (Kitamura et al., 2004; Li et al., 2011; Sun et al., 2012). GSTs have been demonstrated to be related to leaf, flower or fruit pigmentation in fruit crops. For example, *LcGST4* is implicated in anthocyanin transport in litchi pericarp (Hu et al., 2016); In grapevine, *VviGST4* is required for anthocyanin and PA accumulation, whereas *VviGST3* is only involved in PA transport (Pérez-Díaz et al., 2016); In strawberry, *RAP* is required for fruit and foliage anthocyanin production (Luo et al., 2018); The apple fruit anthocyanin sequestration is mediated by *MdGSTF6* (Jiang et al., 2019); *PpGST1* is involved in vacuolar accumulation of anthocyanins in peach fruit (Zhao et al., 2020).

Chinese bayberry (*Morella rubra*, also as *Myrica rubra* previously) is an evergreen fruit tree originated in China, and has since been brought to the United States, Australia, and Japan (Jia et al., 2015, 2019). It has developed into a significant economic fruit crop in southern China. The hue of the Chinese bayberry fruit, which ranges from red to dark purple for most cultivars, is a critical indicator of maturity and quality, and the fruit color in this plant is governed by the presence of anthocyanins. Chinese bayberry fruit is a rich source of anthocyanins and all anthocyanins containing fruit tissues in Chinese bayberry are edible, which shapes this plant a good example for investigating the mechanisms underpinning anthocyanin accumulation. Anthocyanin biosynthesis and transcriptional regulation mechanism in Chinese bayberry have been explored in our previously works (Niu et al., 2010; Liu et al., 2013a,b), however, the mechanism of anthocyanin vacuolar segregation in Chinese bayberry is still poorly understood.

In the present study, a Phi class GST member *MrGST1* was differently expressed in various tissues and cultivars and the expression level was positively correlated with anthocyanin content. *MrGST1* was functionally characterized to participate in anthocyanin accumulation and can be activated by MrMYB1.1. The study provides new insight into mechanisms for pigmentation and its regulation in this plant.

## Materials and Methods

### Plant Materials

The Chinese bayberry (*M. rubra*) cultivar (cv.) “Biqi” samples were collected from a commercial orchard located in Lanxi County, Zhejiang Province, China. Young leaf (YL), mature leaf (ML), young stem (YS), mature stem (MS), flower (F) as well as fruit at six developmental stages were collected. The developmental stages S1, S2, S3, S4, S5, and S6 represents 60, 67, 74, 78, 82, and 86 days after full-bloom (DAFB), respectively. Ripe fruit of other 11 Chinese bayberry cultivars were obtained from the variety repository of Fruit Research Institute in Fujian province, China, including “Baxiandao,” “Dongkui,” “Fenhong,” “Fugongyihao,” “Heijing,” “Longhaishuijing,” “Luozi,” “Ruansianhaibian,” “Shuijing,” “Tezaomei,” and “Wumei.” Three biological replicates were collected, with each replicate including 20 fruit, five fruit, and 30 g for fruit at early developmental stages (S1 and S2), fruit at late developmental stages (from S3 to S6) and other tissues, respectively. All samples were immediately frozen in liquid nitrogen and kept at −80°C for subsequent analysis.

### Anthocyanin Extraction and HPLC Analysis

Anthocyanin extraction and quantification were carried out as reported previously (Zhao et al., 2017). Briefly, extraction was carried out using 2 ml 0.05% HCl in methanol with 100 mg sample powder incubated at 4°C for 12 h and the supernatant obtained by centrifugation. All samples were extracted third times, the supernatant was combined and dried evaporatively using a rotary evaporator. One milliliter 1 M HCl in methanol was added to the residue and filtered with a 0.22 μm Millipore membrane. High-performance liquid chromatography (HPLC) analysis was conducted using a Waters Alliance 2695 system (Waters Corp., United States) equipped with a reverse-phase C_18_ column (4.6 × 250 mm, 5 μm; YMC Co., Ltd., Japan). Chromatographic conditions were as described by Cheng (Cheng et al., 2014). Cyanidin-3-*O*-glucoside (C3G), as the high predominant anthocyanin component, by as much as 97% of total anthocyanins, in Chinese bayberry (Fang et al., 2006), was identified based on its retention time and absorbance at 520 nm and quantified by comparison with its authentic standard curve. Total anthocyanin content in transiently transformed tobacco leaves was presented as C3G equivalent and was calculated by comparison with C3G authentic standard curve based on peak areas recorded at 520 nm.

### RNA Extraction and Real-Time Quantitative PCR Analysis

Total RNA was extracted from Chinese bayberry fruit tissues using a modified cetyltrimethylammonium bromide (CTAB) method (Shan et al., 2008) and from Arabidopsis using the TRIzol Reagent Kit (Ambion, Hopkinton, MA, United States). HiScript® II Q Select RT SuperMix (Vazyme, Nanjing, China) was used to remove genomic DNA contamination from total RNA and to synthesis the first-strand cDNA following the manufacturer’s instructions. RT-qPCR analyses were conducted with ChamQ Universal SYBR qPCR Master Mix (Vazyme, Nanjing, China) in a CFX96 instrument (Bio-Rad, CA, United States) according to the procedure provided by the manufacture. *MrACT* (GenBank accession No. GQ340770) and *ATACT2* (GenBank accession No. AT3G18780) were used as internal controls to normalize the expression of Chinese bayberry and Arabidopsis target genes, respectively. Supplementary Table S1 contains the primer sequences for RT-qPCR.

### Genes Isolation, Promoter Cloning, and Plasmid Construction

The full-length coding sequences (CDS) of all the genes utilized in this study were amplified from the “Biqi” cDNA and cloned into the pEASY-Blunt Simple Cloning Vector (TranGen, Beijing, China) for sequencing. A modified CTAB protocol (Chen et al., 2004) was used to isolate genomic DNA from young Chinese bayberry cultivar “Biqi” leaves. The promoter fragment of *MrGST1* (2.2 kb) was amplified from “Biqi” genomic DNA and the sequence verified as previously described.

For overexpression and dual-luciferase assays, the CDS of *MrMYB1.1* (named as *MrMYB1* previously in literatures; GenBank accession No. GQ340767), *MrbHLH1* (GenBank accession No. JX629461), and *MrGST1* were inserted into the pGreenII 0029 62-SK vector to generate *MrMYB1.1*-SK, *MrbHLH1*-SK and *MrGST1*-SK, respectively. The promoter of *MrGST1* was inserted into the pGreen II 0800-LUC vector to generate *MrGST1*-LUC. Eight MYB binding sites (MBS) of *MrGST1* promoter were mutated one by one with the Fast Mutagenesis System Kit (TransGen) and then inserted into the pGreen II 0800-LUC vector to generate *MrGST1mx*-LUC (x stands for an integer from 1 through 8). The GenePulser Xcell™ Electroporation Systems (Bio-Rad, Hercules, CA, United States) were used to electroporate all constructs into *Agrobacterium tumefaciens* strain GV3101 (MP90).

For yeast one-hybrid assay, the sequence of the *MrGST1* promoter as well as a promoter consisting of three tandem repeats of MBS4 motif were inserted into the pAbAi vector to generate *proMrGST1*-pAbAi and *pro*3 × MBS4-pAbAi, respectively, and CDS of *MrMYB1.1* was inserted into the pGADT7 vector to generate MrMYB1.1-pGADT7.

Supplementary Table S2 contains the PCR primer sequences used to generate vectors.

### Stable Transformation of Arabidopsis

The Arabidopsis transparent *testa19* (*tt19*) mutant (SALK_105779) in the Columbia genetic background was purchased from The Arabidopsis Information Resource (TAIR). A floral-dip method was applied to transform Arabidopsis (Clough and Bent, 1998). Four-week-old seedlings of *tt19* mutant were infiltrated with the *A. tumefaciens* (GV3101) suspension carrying the *MrGST1*-SK construct using the T_1_ transgenic seedlings were screened on 1/2 MS (Murashige and Skoog) medium supplemented with 50 mg/ml kanamycin and 25 mg/ml meropenem.

### Transient Ectopic Expression in *Nicotiana tabacum* Leaves

Transient ectopic expression assays in *Nicotiana tabacum* leaves was performed using the *A. tumefaciens* infiltration (GV3101) with the method previously reported (Sainsbury et al., 2009). Three biological replicates, with every six tobacco plants in each replicate, were set for transient expression analysis. For phenotype identification, the infiltrated plants were put in a growth chamber (25°C, 75% humidity, 16 h light and 8 h dark). One week after infiltration, digital photographs of the color phenotype were taken.

### Yeast One-Hybrid Assay

Yeast one-hybrid assay was conducted to investigate the individual interactions between MrMYB1.1 and *MrGST1* promoter according to the Matchmaker^@^ Gold Yeast One-Hybrid Library Screening System (Clontech) manufacturer’s protocol. Briefly, *proMrGST1*-pAbAi construct was inserted into the genome of the Y1HGold yeast strain. Y1HGold *proMrGST1*-pAbAi strain was tested on synthetic dextrose medium without uracil (SD/-Ura) agar plate to evaluate the background *aureobasidin A resistance* (*AbA^r^*) expression level. After determining the minimal inhibitory concentration of AbA for the Y1HGold *proMrGST1*-pAbAi strains, the pGADT7 and MrMYB1.1-pGADT7 constructs were transformed into the Y1HGold *proMrGST1*-pAbAi strain and screened on synthetic dextrose medium without leucine but supplemented with AbA (SD/−Leu/+AbA) agar plate. All transformations and screenings were carried out for at least three times.

### Dual-Luciferase Assay

Dual luciferase assays were performed in *N. benthamiana* leaves following a previously reported procedure (Zeng et al., 2015). *Agrobacterium tumefaciens* cultures (strain GV3101) containing the pGreen II 0029 62-SK (SK) vector (*MrMYB1.1*-SK, *MrbHLH1*-SK, and *MrGST1*-SK) and the pGreen II 0800-LUC (LUC) vector (*MrGST1*-LUC or *MrGST1mx*-LUC) were combined in a 10:1 (v/v) ratio in MMA buffer (10 mM MES, pH 5.6, 10 mM MgCl_2_, 150 μM acetosyringone), and were then injected into *N. benthamiana* leaves. The *A. tumefaciens* cultures with empty pGreenII 0029 62-SK vector (SK) served as a control. The transcription factor (*MrMYB1.1*, *MrbHLH1*)-promoter (*MrGST1*) interaction was examined after 3 days of infiltration, using dual-luciferase reagents (Promega, Madison, WI, United States) to compare the level of firefly luciferase (LUC) and renilla luciferase (REN) intensities. The results were calculated based on the data obtained from three independent experiments, each of which contained six replicate reactions.

### Bioinformatic Analysis

GSTs Hidden Markov Model (HMM) profile downloaded from Pfam database^1^ was used to identify GST genes in Chinese bayberry genome (Jia et al., 2019). The ClustalW tool was used to align the protein sequences of Chinese bayberry and known GSTs of other plants. Phylogenetic tree was generated using the Maximum Likelihood method with 1,000 bootstrap replicates in MEGA 11 software (Tamura et al., 2021). The *cis*-acting elements were predicted in PlantCARE^2^ and PLACE (Plant *cis*-acting Regulatory DNA Elements)^3^ databases.

### Statistical Analysis

The IBM SPSS Statistics for Windows, version 23.0 (IBMCorp., Armonk, N.Y., United States) was used for the data analysis. The Pearson correlation coefficient (*R*) as used to determine the strength of the association between variables (i.e., subjective vs. quantitative). The coefficient of determination (*R*^2^) and the significance of the relationship between variables was indicated by probability levels. To characterize the relationship between variables, a linear regression model was applied to the obtained data.

## Results

### Identification and Characterization of *MrGST1* in Chinese Bayberry

**Figure 1:**
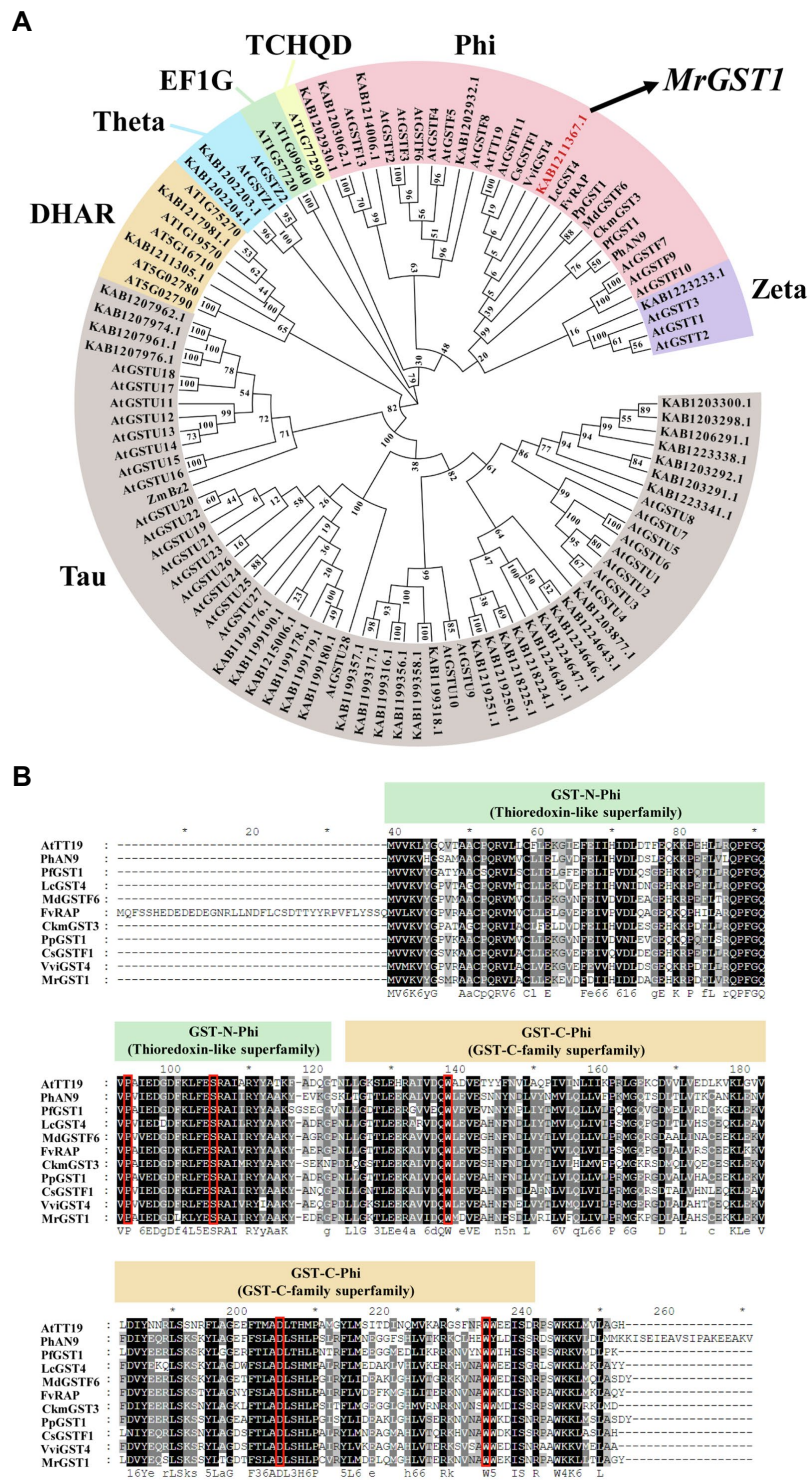
Identification of anthocyanin-related glutathione *S*-transferases (GST) transporter from the Chinese bayberry (*Morella rubra*) genome. **(A)** Phylogenetic analysis of 105 GSTs based on deduced amino acid sequence, including 42 from Chinese bayberry, with names beginning with KAB, 53 from Arabidopsis, with names beginning with AT or At, as well as 10 from other plants with function characterized as anthocyanin transport related. Gene IDs are shown for genes from Chinese bayberry. The full-length amino acid sequences of GSTs family members of Arabidopsis were downloaded from The Arabidopsis Information Resource (TAIR, https://www.arabidopsis.org/). The accession numbers in NCBI are shown for others: PfGST1 (*Perilla frutescens*, AB362191), CkmGST3 (*Cyclamen*, AB682678), PhAN9 (*Petunia hybrida*, Y07721), VviGST4 (*Vitis vinifera*, AAX81329), CsGSTF1 (*Camellia sinensis*, ABA42223), LcGST4 (*Litchi chinensis*, KT946768), FvRAP (*Fragaria ananassa*, gene31672), PpGST1 (*Prunus persica*, Prupe.3G013600), MdGSTF6 (*Malus domestica*, MD17G1272100), ZmBz2 (*Zea mays*, NP_001183661.1). The member KAB1211367.1 is denoted as MrGST1 in this study. **(B)** Protein sequence alignment of MrGST1 (KAB1211367.1) and anthocyanin-related GSTs from other dicotyledonous plants. Numbers in the alignments showed amino acid positions. Red boxes denoted amino acid residues previously identified as high-homology locations in the GST family (Alfenito et al., 1998).

To screen Chinese bayberry *GST* genes associated with anthocyanin accumulation, HMM profiles of the N-terminal conserved domain (PF02798) and the C-terminal conserved domain (PF00043) were used to screen the Chinese bayberry genome. As a result, a total of 42 *GST* gene family members were identified. To elucidate the evolutionary relationship among the Chinese bayberry GST proteins, a phylogenetic tree was constructed using their protein sequences with 53 GSTs from Arabidopsis (Dixon et al., 2002a,b; Sappl et al., 2009) and 10 GSTs from other plants with function characterized as anthocyanin transport related (Figure 1A). As shown in the phylogenetic tree, KAB1211367.1 was the closest homologs of MrGSTs in the Phi subfamily which comprises anthocyanin-related GSTs in other plants like AtTT19 (*Arabidopsis thaliana*; Sun et al., 2012), PfGST1 (*Perilla frutescens*; Yamazaki et al., 2008), CkmGST3 (*Cyclamen persicum*; Kitamura et al., 2012), PhAN9 (*Petunia hybrida*; Alfenito et al., 1998), VviGST4 (*Vitis vinifera*; Pérez-Díaz et al., 2016), CsGSTF1 (*Camellia sinensis*; Wei et al., 2019), LcGST4 (*Litchi chinensis*; Hu et al., 2016), FvRAP (*Fragaria ananassa* Sonn.; Luo et al., 2018), PpGST1 (*Prunus persica* L. Batsch; Zhao et al., 2020), MdGSTF6 (*Malus* × *domestica* Borkh.; Jiang et al., 2019). Physical location of *MrGST1* is on chromosome 6 of Chinese bayberry genome, same with two previously reported fruit coloration related transcription factor genes *MrMYB1.1* (Cao et al., 2021) and *MrMADS01* (Zhao et al., 2019) in a linkage of around 1 Mb. The full-length *MrGST1* coding sequence was amplified from “Biqi” cDNA library. The *MrGST1* encoded a putative protein of 215 amino acids with a predicted molecular weight of 24.54 kDa and an isoelectric point of 6.09. An alignment of MrGST1 protein with anthocyanin-related GSTs identified in other dicotyledonous plants indicated that all of these GSTs had the conversed GST-N-Phi (Thioredoxin-like superfamily), GST-C-Phi (GSTC-family superfamily) domains, and high-homology sites (indicated as red boxed) in the GST family (Figure 1B).

### Expression of *MrGST1* Correlated Well With Anthocyanin Accumulation in Chinese Bayberry

**Figure 2:**
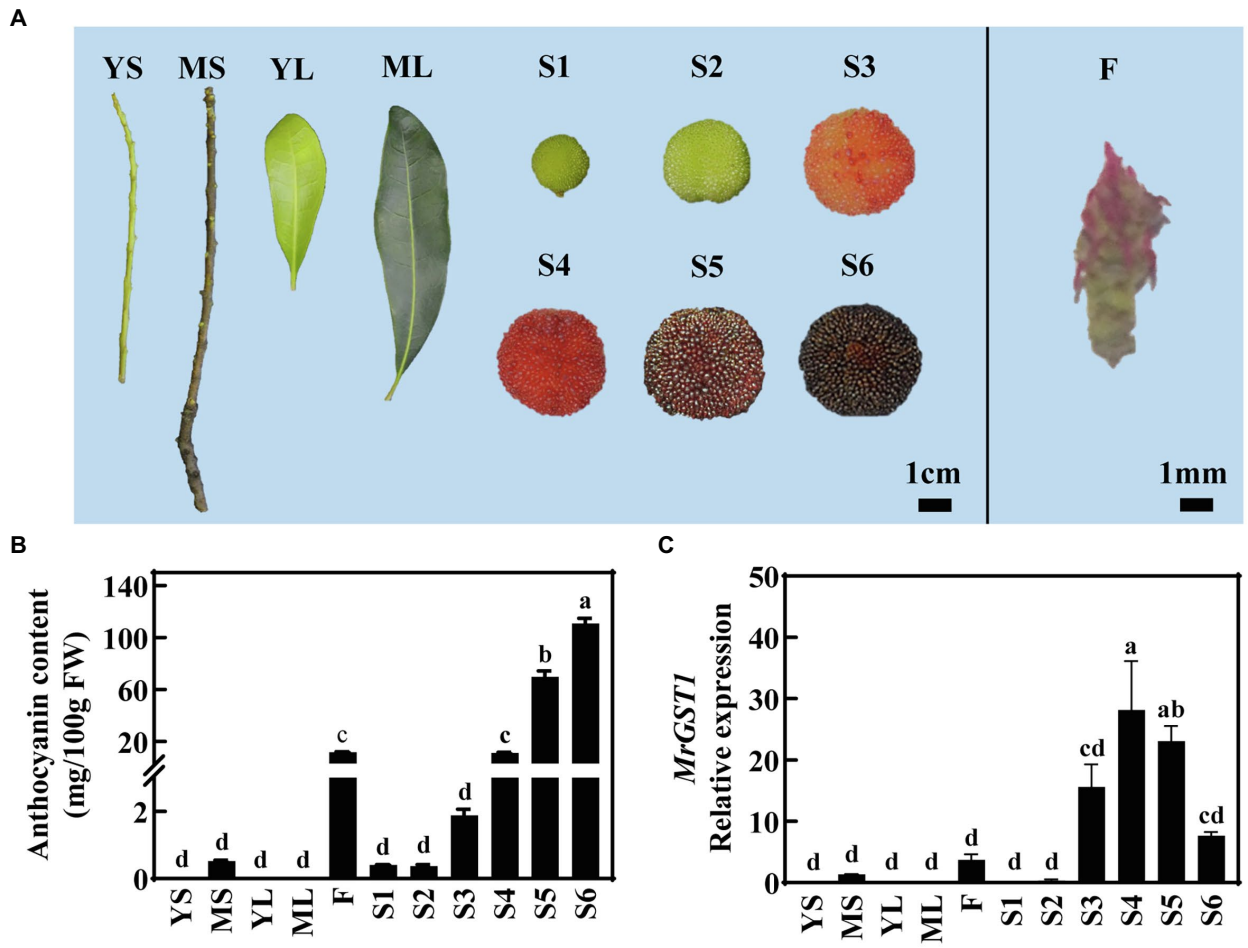
Expression of *MrGST1* related to anthocyanin accumulation in different tissues and fruit at different developmental stages of “Biqi” Chinese bayberry. **(A)** Photo of different tissues, including YS (young stems), MS (mature stems), YL (young leaves), ML (mature leaves), S1 (fruit at 60 days after full-bloom, DAFB), S2 (67 DAFB), S3 (74 DAFB), S4 (78 DAFB), S5 (82 DAFB), S6 (86 DAFB), and F (Flowers). **(B)** Anthocyanin content. **(C)** *MrGST1* (KAB1211367.1) expression levels. Data were means (±SE) from three independent biological replicates. Same letter indicates no significant difference (*p* < 0.05).

To ascertain whether *MrGST1* contributed to anthocyanin accumulation in Chinese bayberry, various tissues and different fruit development stages of “Biqi” were sampled (Figure 2A) for quantification of anthocyanin content. The leaves, young stem (YS), and fruit at early developmental stages (S1 and S2) were green, the mature stem (MS) was brown, and the flower was red. Slight red pigmentation appeared in fruit at S3 and gradually deepened as fruit ripens. The content of C3G, the high predominant anthocyanin component in Chinese bayberry (Fang et al., 2006), was under detectable in leaves and YS, showed a low concentration in MS and flower, and increased during late fruit developmental stages reaching up to 110.85 mg/100 g FW (fresh weight) at S6, consistent with visual appearances (Figures 2A,B).

The relative expression of *MrGST1* was determined in various tissues and in fruit at different developmental stages of “Biqi.” *MrGST1* expression was observed in MS, flower and fruit at late developmental stages but was undetectable in YS and leaves, which was consistent with the anthocyanin accumulation profile (Figures 2B,C). With fruit at six developmental stages, the linear-regression relationship among transcription level of 11 anthocyanin-related genes, including *MrGST1*, were established (Supplementary Table S3). Expression of *MrGST1* showed a positive correlation with most biosynthetic genes except for *MrCHI*, as well as with *MrMYB1.1* (*R* = 0.714, *p* < 0.01) but not *MrbHLH1* or *MrWD40-1*. These results suggested that coordinated expression of biosynthetic genes, transport gene *MrGST1* as well as upstream transcription factor gene *MrMYB1.1* probably plays an important role in the coloration during Chinese bayberry fruit ripening.

**Figure 3:**
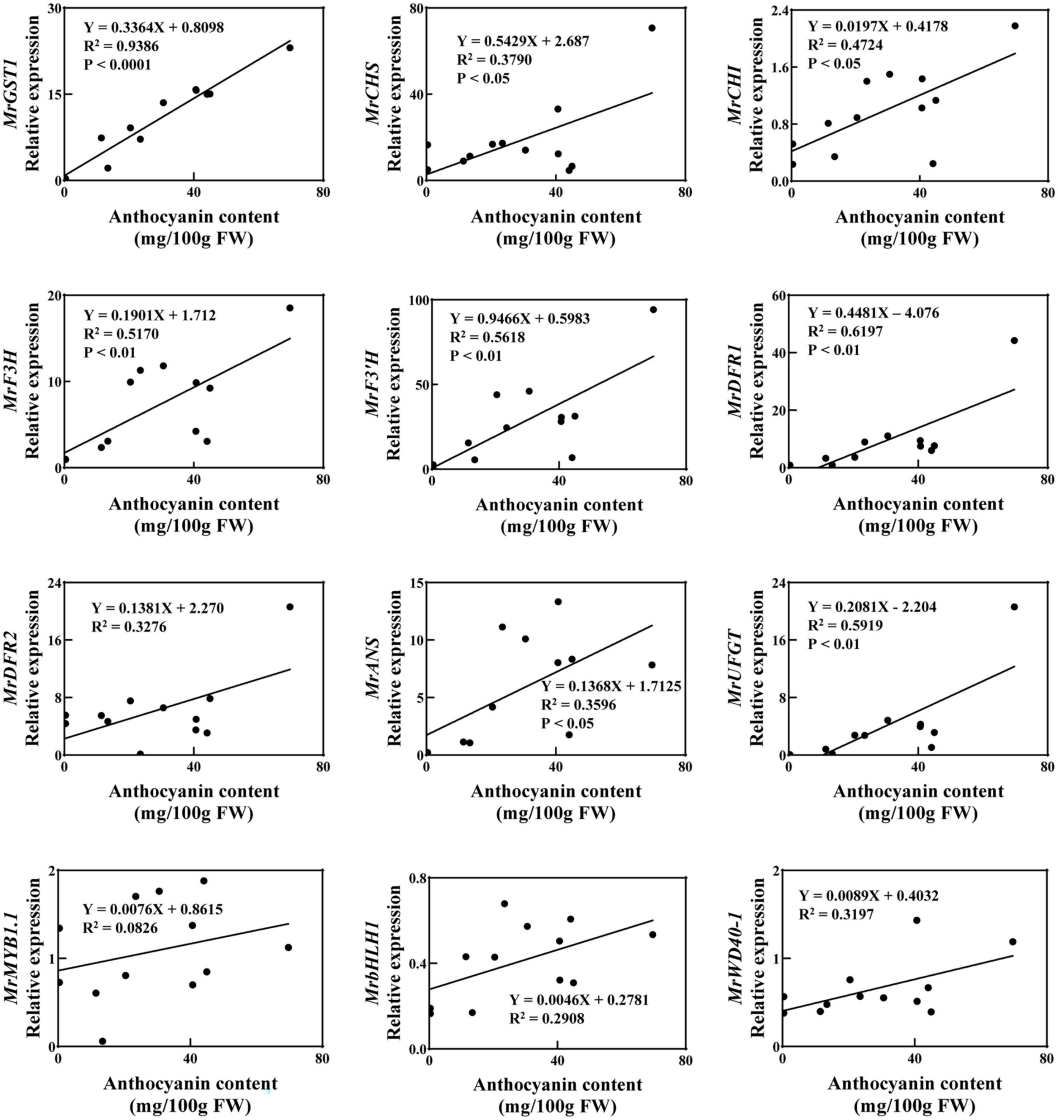
Correlation between the anthocyanin content and expression of biosynthetic genes, transcription factors and *MrGST1* of 12 Chinese bayberry cultivars fruit at 86 DAFB. Data were means (±SE) from three independent biological replicates.

To further explore the relationship between gene expression and anthocyanin content, with ripe fruit of all 12 cultivars, correlation analysis was carried out between anthocyanin content and transcript levels of *MrGST1*, as well as anthocyanin biosynthetic genes and transcription factors (Figure 3). These cultivars varied in their coloration from being totally white to deeply red. The linear-regression relationship between anthocyanin content and transcription level of 12 anthocyanin-related genes were different. Expression of *MrGST1* was highest positively correlated with anthocyanin content (*R*^2^ = 0.9386, *p* < 0.0001) than all other genes. Expression of biosynthetic genes, only except for *MrDFR2*, were significantly positively correlated with anthocyanin content. However, significant linear relationships were not discovered between anthocyanin content and expression of transcription factors.

Additionally, correlation analysis between the transcription levels of *MrGST1* and the other 11 anthocyanin-related genes in these 12 cultivars was performed (Supplementary Table S4). Expression of *MrGST1* showed a positive correlation with all biosynthetic genes significantly, whereas significant linear relationships were not discovered between expression of *MrGST1* and the transcription factors. Taken together, these results indicated that *MrGST1* plays a critical role in anthocyanin accumulation in Chinese bayberry.

### *MrGST1* Is the Ortholog of Arabidopsis *TT19*

**Figure 4:**
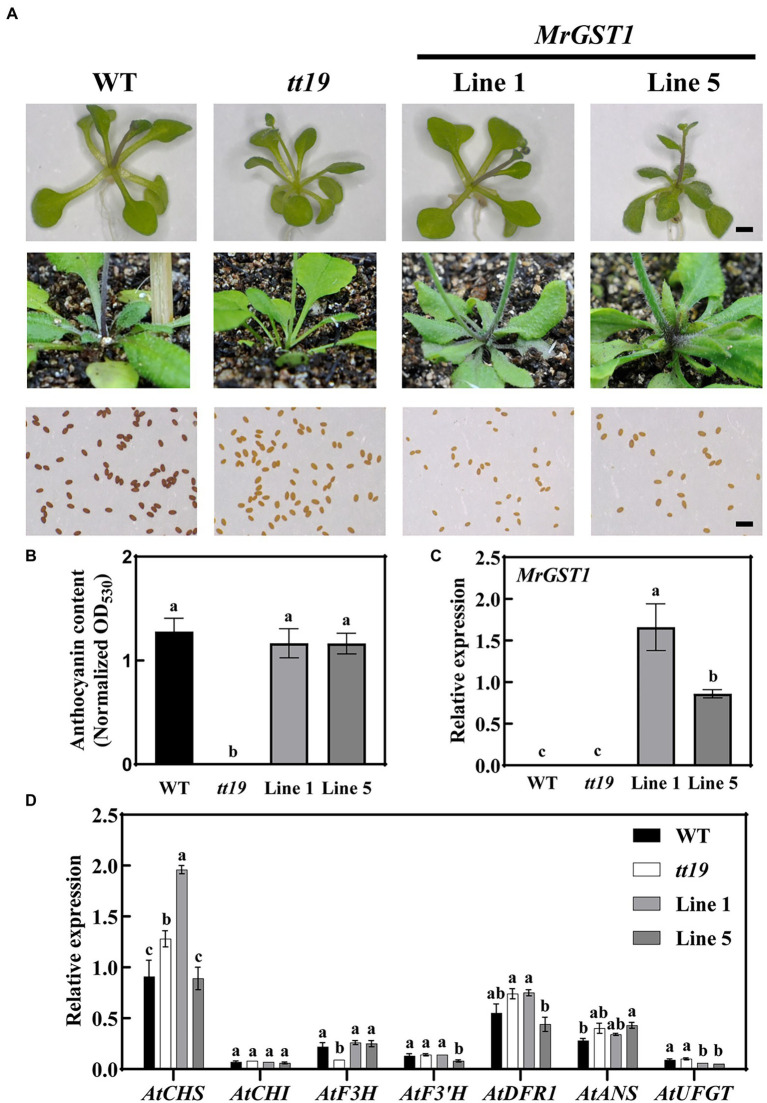
Complementation of *tt19* mutant with functional *MrGST1* (glutathione *S*-transferase). **(A)** Phenotypes of the Arabidopsis wild type (WT), mutant (*tt19*), and transgenic lines (*35S::MrGST1*/*tt19*, Line 1 and Line 5). Pictures of the seedlings, the base of the rosette and fresh seeds were shown. Black bar: 1 mm. **(B)** Anthocyanin content. **(C, D)** Expression of *MrGST1* **(C)** and anthocyanin-related genes **(D)** of base portion of the rosette and stems. Data were means (±SE) from three independent biological replicates. Same letter indicates no significant difference (*p* < 0.05).

Arabidopsis wild type harbors the functional TT19, which serves as a carrier to transport anthocyanin from the cytosol to tonoplasts and accumulate anthocyanins at the basal portion of stem and rosette (Sun et al., 2012). *MrGST1* is the homolog of Arabidopsis *TT19*. To investigate the function of *MrGST1* in anthocyanin transport, *35S::MrGST1* was transformed into the Arabidopsis mutant *tt19*, a knockout mutant of the anthocyanin transporter *AtTT19*. Purple pigmentation at the basal portion of the stem and rosette of Arabidopsis wild type (WT) was observed, whereas the mutant tt19 did not. This is consistent with previous studies (Sun et al., 2012; Luo et al., 2018; Jiang et al., 2019; Zhao et al., 2020). The *35S::MrGST1*/*tt19* transgenic plants (Line 1 and Line 5) recovered the purple pigmentation phenotype (Figure 4A). The content of anthocyanin at the basal portion of stem of *tt19* and transformants was consistent with visual appearance (Figure 4B). High expression levels of *MrGST1* in the transgenic lines were validated by qRT-PCR (Figure 4C). However, transgenic plants harboring *MrGST1* failed to complement the brown color of seed coats of *tt19*, pointing to the possibility that *MrGST1* performs different functions during seed-coat pigmentation from *TT19* and did not participate in PA accumulation (Figure 4A). Expression levels of Arabidopsis genes involved in anthocyanin production were also examined using quantitative real-time PCR (Figure 4D). It was found that the expression levels of genes involved in anthocyanin biosynthesis did not vary significantly among Arabidopsis wild type, mutant *tt19*, and *35S::MrGST1*/*tt19* transgenic plants, indicating that variations in anthocyanin content were not attributable to biosynthetic genes and *TT19*, or *GST*, gene performs a role downstream of the anthocyanin biosynthesis pathway, as previously reported (Kitamura et al., 2004; Sun et al., 2012; Luo et al., 2018; Jiang et al., 2019; Zhao et al., 2020). This result was consistent with the findings described by Sun et al. (2012), where *tt19* has no effect on the level of expression of genes involved in anthocyanin biosynthesis.

### Transient Ectopic Co-expression of *MrGST1* With *MrMYB1.1* and *MrbHLH1* Results in Elevated Anthocyanin Accumulation in *Nicotiana tabacum* Leaves

**Figure 5:**
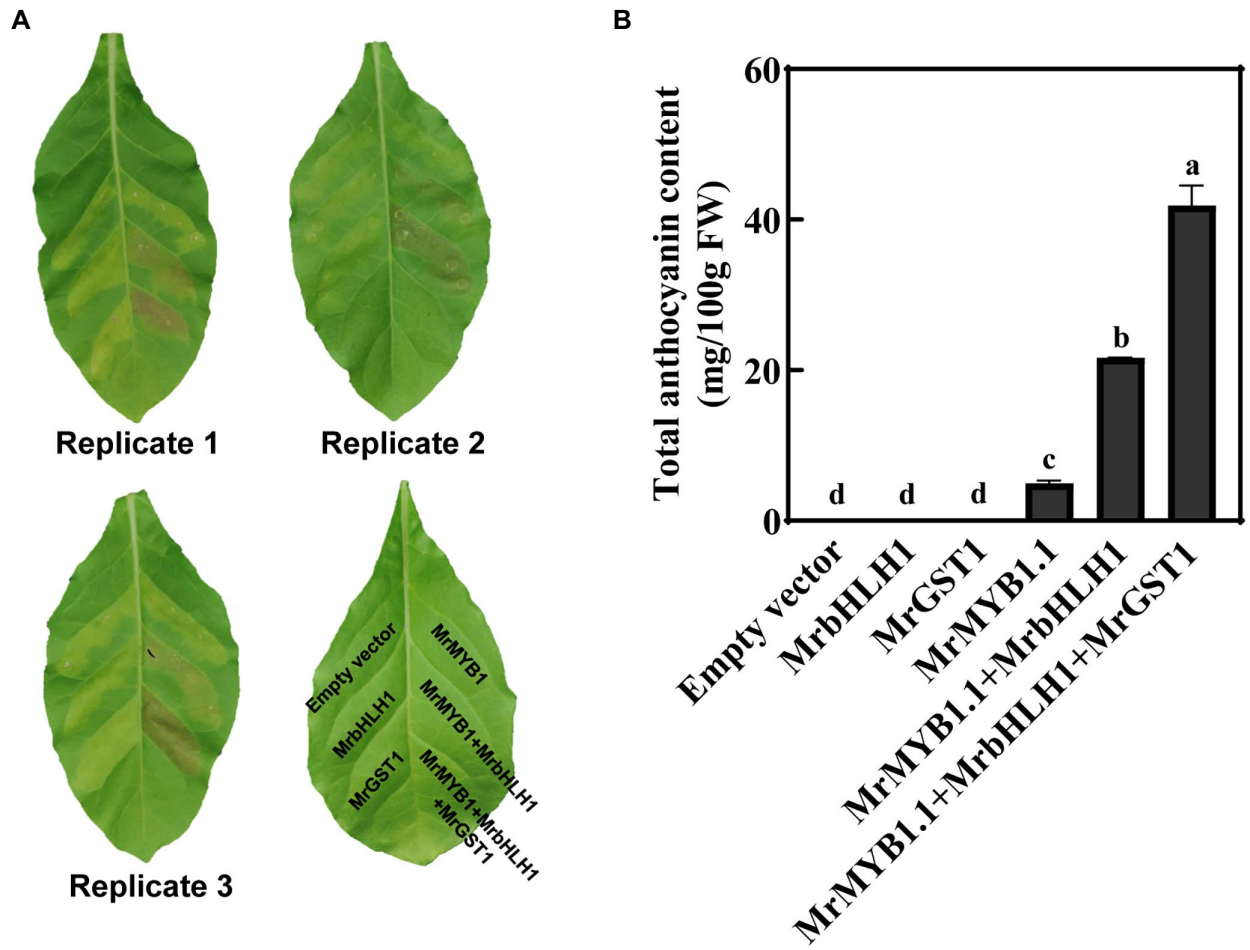
Ectopic transient expression of *MrGST1* in *Nicotiana tabacum* leaves. **(A)** Digital images of *N. tabacum* leaves were taken at 5 days after infiltration. **(B)** Total anthocyanin content of transformed leaves at infiltration sites. Anthocyanins were quantified as cyanidin-3-*O*-glucoside (C3G) equivalents. Data were means (±SE) from three independent biological replicates. Same letter indicates no significant difference (*p* < 0.05).

To further evaluate the contribution of *MrGST1* in anthocyanin accumulation, transient ectopic expression in *N. tabacum* leaves was performed (Figure 5A). Red patches appeared at 3 days after infiltrations at the injected areas after transformation with *35S::MrMYB1.1* + *35S::MrbHLH1* and *35S::MrMYB1.1*  +  *35S::Mr bHLH1*  +  *35S::MrGST1*, while no obvious pigmentation appeared when *35S::MrMYB1.1*, *35S::MrbHLH1*, and *35S::MrGST1* alone was injected. Anthocyanin content was detected in *35S::MrMYB1.1* injected areas but not *35S::MrbHLH1* and *35S::MrGST1* injected areas (Figure 5B). Pigmentation was obvious when leaves were co-infiltrated with *35S::MrMYB1.1* and *35S::MrbHLH1*, and a much more intense pigmentation was detected when co-infiltrated with *35S:: MrMYB1.1*, *35S:: MrbHLH1* and *35S::MrGST1* (Figure 5A). The data on anthocyanin content were well matched with the visual outcomes. The content was 93% higher in the *35S::MrMYB1.1*  +  *35S:: MrbHLH1*  +  *35S::MrGST1* injected areas than *35S::Mr MYB1.1* + *35S::MrbHLH1* injected areas (Figure 5B), implicating that *MrGST1* is a rate-limiting step for anthocyanin accumulation.

### Analysis of *cis*-Elements in the *MrGST1* Promoter

The promoter sequence up to 2,229 bp upstream of the start codon ATG of *MrGST1* was cloned and analyzed using the PlantCARE and PLACE online tools. Multiple transcription factor (TF) binding sites were discovered in the *MrGST1* promoter, including DOF, ARR, MYB, MYC, RAV, and WRKY (Supplementary Table S5). Notably, eight MYB binding sites (MBSs) were observed in *MrGST1* promoter region. Additionally, various responsive elements in the *MrGST1* promoter were identified, including light-responsive elements (G-box, AT1-motif, Box4, GT-1 motif and I-box), temperature responsive elements (LTRECOREATCOR15), hormone-responsive elements such as auxin-responsive elements (AuxRE, TGA-element, ARR), gibberellin-responsive elements (P-box, GARE-motif, TATC-box), ethylene-responsive elements (ERE), ABA-responsive elements (DPBFCOREDCDC3, ABRE, ACGTT box), and other stress-responsive elements (GC-motif), demonstrating that numerous environmental and physiological variables including light, temperature, hormones, and abiotic stressors, may influence the transcription of *MrGST1*.

### Identification of the Critical MBS for the Activation of *MrGST1* Expression by MrMYB1.1

**Figure 6:**
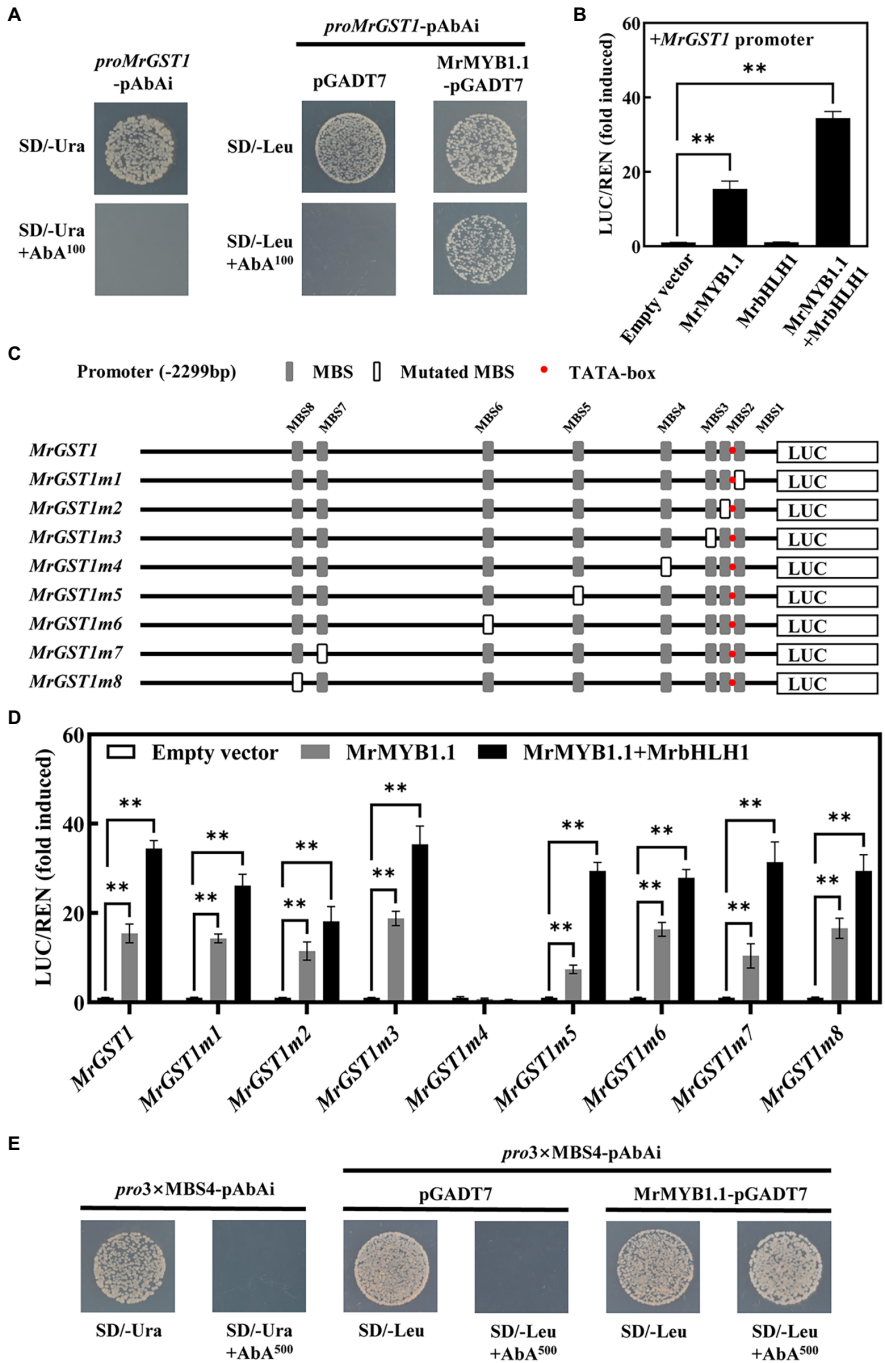
Activation of the *MrGST1* by MrMYB1.1. **(A)** Yeast one-hybrid interaction between MrMYB1.1 and *MrGST1* promoter. **(B)** Effects of MrMYB1.1 (+/− MrbHLH1) on the promoter activity of *MrGST1* measured by dual-luciferase assays. **(C)** Schematic diagrams of the *MrGST1* promoter with eight MYB binding site (MBS) motif mutations. **(D)** Effects of MrMYB1.1 (+/− MrbHLH1) on the activity of original and mutated promoters of *MrGST1* measured by dual-luciferase assays. **(E)** Yeast one-hybrid interaction between MrMYB1.1 and three tandem repeats of MBS4 motif of *MrGST1* promoter. Data were means (±SE) from three independent biological replicates. Asterisks (^**^) indicate significant differences at *p* < 0.05 level.

Yeast one-hybrid assay was conducted to explore whether MrMYB1.1 can bind to the promoter of *MrGST1*. As shown in Figure 6A, the minimal inhibitory concentration of AbA for the Y1HGold *proMrGST1*-pAbAi strains was 100 ng/ml. After transforming the pGADT7 and MrMYB1.1-pGADT7 constructs into Y1HGold *proMrGST1*-pAbAi strains, the yeast cells harboring MrMYB1.1-pGADT7 could grow on the SD/−Leu/AbA^100^ agar plate while the control pGADT7 could not. These data indicated that MrMYB1.1 physically binds to the *MrGST1* promoter (Figure 6A). To explore whether MrMYB1.1 affects the transcriptional activity of the *MrGST1* promoter, we conducted dual luciferase assays. When compared to the control, MrMYB1.1 had considerable transactivation effects on the promoter of *MrGST1*, with a 15.4-fold increase in activity (Figure 6B). When MrMYB1.1 was co-infiltrated with MrbHLH1, a 34.4-fold increase in the expression of the *MrGST1* promoter was detected. However, MrbHLH1 infiltration alone exhibited no activation effect. There are eight MYB binding sites in the *MrGST1* promoter (2,229 bp) based on the *cis*-elements analysis (Figure 6C). Subsequent tests were conducted to determine the essential MBS elements in the promoter of *MrGST1*. Original and mutated sequences of eight MYB binding sites one by one were listed in Supplementary Table S6. When the fourth MBS from the ATG start site, MBS4 (TACCAACC), was mutated to TTGGATCG in *MrGST1* promoter (*MrGST1m4*, Figure 6D), transactivation activity of MrMYB1.1 and MrMYB1.1 + MrbHLH1 was abolished, with activity reduced by 95% and 98%, respectively (Figure 6D). The activity of other mutated *MrGST1* promoters containing non-mutated MBS4 but with mutation in one of the other seven MBSs was still greatly boosted by MrMYB1.1 and MrbHLH1 (Figure 6D). To validate the interaction between MrMYB1.1 and MBS4 motif, three tandem repeats of MBS4 was cloned into the pAbAi vector and yeast one-hybrid assay was conducted. As shown in Figure 6E, after transforming the pGADT7 and MrMYB1.1-pGADT7 constructs into Y1HGold *pro*3 × MBS4-pAbAi strains, the yeast cells harboring MrMYB1.1-pGADT7 could grow on the SD/−Leu/AbA^500^ agar plate while the control pGADT7 could not. The results showed that MrMYB1.1 recognized the MBS4 and can bind to the MBS4 motif to activate the *MrGST1* expression.

## Discussion

### *MrGST1* Plays a Crucial Role in Regulating Anthocyanin Transport in Chinese Bayberry

Anthocyanins are important for fruit not only because of providing vibrant hues, but being helpful to human health as well. As a result, there is a great deal of studies interested in controlling the anthocyanin accumulation. Anthocyanins are synthesized in the cytosol and eventually accumulated in vacuole. Over the last few decades, the processes underpinning the intracellular anthocyanin transport have been largely unraveled. The first GST to be implicated in anthocyanin accumulation is a Tau class protein from maize (*Zea mays*), which is localized to the bronze2 (*bz2*) locus and is found to be involved in the pigmentation of red and purple kernels (Marrs et al., 1995). GSTs discovered to participate in anthocyanin transport in dicotyledonous plants over the next two decades are all members of the Phi family, including *PhAN2* (Alfenito et al., 1998), *AtTT19* (Kitamura et al., 2004), *PfGST1* (Yamazaki et al., 2008), *CkmGST3* (Kitamura et al., 2012), *VviGST4* (Pérez-Díaz et al., 2016), *LcGST4* (Hu et al., 2016), *FvRAP* (Luo et al., 2018), *MdGSTF6* (Jiang et al., 2019) and *PpGST1* (Zhao et al., 2020). In this study, MrGST1 involved in anthocyanin transport in Chinese bayberry was identified. *MrGST1* transcript levels was consistent with anthocyanin content of various tissues and ripe fruit of different cultivars, and had a highest positive correlation with anthocyanin content than other anthocyanin-related genes, indicating that it may be the member of GST involved in anthocyanin transport.

In other plants, through functional complementation with various mutants, the role of GSTs in anthocyanin transport has been demonstrated. In maize, the *bz2* mutant accumulates anthocyanin only in the cytosol (Marrs et al., 1995). Petunia *An9* was discovered *in vivo* to conduct comparable roles to *Bz2* (Alfenito et al., 1998). Heterologous expression of *BZ2* and *AN9* restored the anthocyanin accumulation in carnation mutant *fl3* (Larsen et al., 2003). Strawberry mutant *rap* was discovered to have green foliage because of a faulty GST gene (Luo et al., 2018). Anthocyanin-less phenotypes as well as PA-deficient phenotypes in seed coat can be functionally compensated in Arabidopsis *tt19* mutant by homologous overexpression of *VviGST4* and *AcGST1* (Pérez-Díaz et al., 2016; Liu et al., 2019). In this study, *MrGST1* was the only *GST* member capable of complementing the anthocyanin-deficient phenotype of the Arabidopsis *tt19* mutant but not the PA-deficient phenotype, which was identical to the result of *An9* (*Petunia hybrida*; Alfenito et al., 1998), *LcGST4* (*Litchi chinensis*; Hu et al., 2016), *RAP* (*Fragaria ananassa*; Luo et al., 2018), *CsGSTF1* (*Camellia sinensis*; Wei et al., 2019), *MdGSTF6* (*Malus domestica*; Jiang et al., 2019) and *PpGST1* (*Prunus persica*; Zhao et al., 2020) in the *tt19* complementation assay. These results suggested that *MrGST1* participated in anthocyanin transport and may have functions differently from *TT19* during seed-coat coloring.

### Expression of *MrGST1* Is Regulated *via* Multiple Means

Due to the critical role of *MrGST1* in anthocyanin accumulation, several efforts have been conducted to determine the variables controlling *MrGST1* expression. According to the *cis*-acting elements analysis, multiple transcription factor (TF) binding sites were discovered in the promoter of *MrGST1*. MYB transcription factors are of special importance among these TFs since in other plants several studies have demonstrated that the expression of anthocyanin-related *GSTs* is induced by MYB transcription factors. In Arabidopsis, *PAP1* (a R2R3 MYB transcription factor) overexpression results in the upregulation of *TT19*, a *GST* member (Wangwattana et al., 2008). *FvRAP* is significantly induced *via* overexpression of *FvMYB10* in strawberry (Luo et al., 2018). Similar findings were also reported in litchi, tea, apple, kiwifruit, and peach (Hu et al., 2016; Jiang et al., 2019; Liu et al., 2019; Wei et al., 2019; Zhao et al., 2020). In previous studies, MrMYB1.1 was found to be a key transcription factor in the production of anthocyanin in Chinese bayberry (Niu et al., 2010). The higher correlation was observed between expression level of *MrGST1* and downstream structural genes during the fruit development of “Biqi” (Supplementary Table S3) and ripe fruits among different cultivars (Supplementary Table S4). Therefore, we speculated that *MrGST1* might be regulated by MrMYB1.1 transcription factor like anthocyanin biosynthetic genes. Here the data from yeast one-hybrid and dual luciferase assays indicated that MrMYB1.1 directly bind to the *MrGST1* promoter and activated the transcriptional activity of the *MrGST1* promoter, and the transcriptional activity of *MrGST1* promoter was further up-regulated *via* the cooperation between MrMYB1.1 and MrbHLH1. *Cis*-elements analysis revealed that *MrGST1* promoter comprised eight MBSs, with MBS1, MBS2, MBS5, and MBS6 belong to the MBSI type [C(A/C/G/T)GTT(A/G)], MBS2 belongs to the MBSII type [G(G/T)T(A/T)GTT(A/G)], MBS4 and MBS8 belong to the MBS IIG type [(C/T)ACC(A/T)A(A/C)C; Prouse and Campbell, 2012]. It has been proposed that the majority of MYB members of group C bind to MBS IIG and MrMYB1.1 was previously shown to be clustered related with AtMYB75 and AtMYB90 (Cao et al., 2021), both of which belong to the group C (Prouse and Campbell, 2012). In this study, MrMYB1.1 specially recognized the MBS4 (TACCAACC, 348 bp upstream of ATG start site), the fourth MBSs from the ATG start site (Figure 6), consistent with the cases that MYB proteins of group C usually bind to MBS IIG, and typically located around 500 bp upstream of the transcriptional start site (Prouse and Campbell, 2012). Our results revealed that MrMYB1.1 acted as an activator of anthocyanin transport by stimulating *MrGST1* expression.

GSTs from other plants have been demonstrated to be variably modulated by a number of stimuli, consist of abiotic and biotic stresses, GSH, heavy metals, hydrogen peroxide, and plant hormones such as ABA, cytokinins and auxins (Oztetik, 2008; Sappl et al., 2009). In Arabidopsis, auxin stimulates the transcript levels of *GST* genes (Goda et al., 2004). In crop plant rice, *GST*s are differentially expressed under auxin and cytokinin treatments (Jain and Khurana, 2009; Jain et al., 2010). Given that anthocyanin contents are increased in response to various pressures, it is possible that the GSTs implicated in anthocyanin accumulation are likewise sensitive to both internal and external influences. In accordance with this hypothesis, jasmonic acid significantly increases the anthocyanin content of grape cell suspension cultures and expression of all GST genes (Conn et al., 2008). ABA treatment and light dramatically increased the transcription of the litchi GST, *LcGST4* (Hu et al., 2016). UV irradiation was used to increase the amount of *PpGST1* transcripts (Zhao et al., 2017). Here in Chinese bayberry, the expression of *MrGST1* was not always consistent with that of *MrMYB1.1*, for example, among ripe fruit of 12 cultivars, the expression of *MrGST1* was quite loosely correlated with *MrMYB1.1* (*R* = 0.100, Supplementary Table S4). Therefore, further studies on regulation of *MrGST1* expression needs to be pursued beyond MrMYB1.1. Through *cis*-element analysis, numerous light-responsive, temperature-responsive, and hormone-responsive components were discovered in the *MrGST1* promoter (Supplementary Table S5), showing that *MrGST1* expression may be controlled by multiple factors, both of internal and external, which provides clues for further investigation.

## Conclusion

In summary, the Chinese bayberry MrGSTs were characterized in the present study, and MrGST1 was found to be responsible for anthocyanin transport. Additionally, MrMYB1.1 transactivated the *MrGST1* promoter significantly, and MBS4, the fourth MBS from the ATG start site, was identified as the most critical MBS. These results provide insights into the fundamental regulatory mechanism of *MrGST1* and shed light on novel strategies for manipulating anthocyanin accumulation in Chinese bayberry and other fruits.

## Data Availability Statement

The original contributions presented in the study are included in the article/Supplementary Material; further inquiries can be directed to the corresponding author.

## Author Contributions

CX conceived the research plans and supervised the experiments. LX, XH, YZ, ZZ, QL, and QZ performed the experiments and analysis. LX wrote the manuscript. CX and ZG were involved in revising the manuscript. All authors contributed to the article and approved the submitted version.

## Funding

This work was supported by the Natural Science Foundation of Zhejiang Province, China (LZ17C150001), the Natural Science Foundation of Fujian Province, China (2019J01111), and the “5511” Collaborative innovation Project (XTCXGC2021019-GSS01).

## Conflict of Interest

The authors declare that the research was conducted in the absence of any commercial or financial relationships that could be construed as a potential conflict of interest.

## Publisher’s Note

All claims expressed in this article are solely those of the authors and do not necessarily represent those of their affiliated organizations, or those of the publisher, the editors and the reviewers. Any product that may be evaluated in this article, or claim that may be made by its manufacturer, is not guaranteed or endorsed by the publisher.

## Footnotes

^1^ http://pfam.xfam.org/

^2^ http://bioinformatics.psb.ugent.be/webtools/plantcare/html/

^3^ http://www.dna.affrc.go.jp/PLACE/signalscan.html
