# Supplementary material for: An Anthocyanin-Related Glutathione S-Transferase, MrGST1, Plays an Essential Role in Fruit Coloration in Chinese Bayberry (Morella rubra)
Source: Front Plant Sci. 2022 Jun 8;13:903333. doi: 10.3389/fpls.2022.903333 (PMC9213753; doi:10.3389/fpls.2022.903333)
Supplement: Supplementary file 1 [file Table_1.DOCX]

**Table S1** Primers used for Chinese bayberry and Arabidopsis reverse transcription quantitative PCR (qRT-PCR) analysis.

| **Gene** | **Forward primer (5′ to 3′)** | **Reverse primer (5′ to 3′)** | **GenBank No./Source** |
| --- | --- | --- | --- |
| *MrCHS* | AGTTCAAGCGCATGTGTGAC | TGGCAGCTTCTTTGCCTAGT | GQ340759 |
| *MrCHI* | GCCATCGGGGTGTACTTAGA | GTTACCCGATAACGGCAAGA | GQ340760 |
| *MrF3H* | GTCGACATGGACCAGAAGGT | GGAGCAAGAGGGTGATGGTA | GQ340761 |
| *MrF3’H* | ATGAAGCATATGGCCTGACC | CGTAGCAGTAGCACCCACAA | GQ340762 |
| *MrDFR1* | GACAATCAACGGGTTGTTAG | GACTGGCTTTTGGTGCTCTTC | GQ340763 |
| *MrDFR2* | AACGGTGAACGGGGTGTTGA | TACTTTCCTTTGGTGCTCAGA | GQ340764 |
| *MrANS* | CTAGTGGGAAGCTCGAGTGG | GCTAGCGCTCTCAGTTGCTT | GQ340765 |
| *MrUFGT* | TTTCCTCGACCAAACCAGAC | CTTACCTCCCTCCCCATCTC | GQ340766 |
| *MrMYB1.1* | GGTGGTGAAAAAGCTCAACAA | CAGGTTTTCCCCCCATTTTAC | GQ340767 |
| *MrbHLH1* | GGAGGTGAAGAGGGCAATAAA | CACGGCTACTTCTCGATGGTA | JX629461 |
| *MrWD40-1* | CTGTTGAAACACCCGAACTC | AGCGTCAAAGCGTCCGGGTC | Liu et al., 2013a |
| *MrGST1* | CGACCTGCTTGGAAGAAGTTAC | GAAAACCAAGGAAGATAGTGGAAAG | This study |
| *MrACT* | TGGATTTGCTGGAGACGATG | CTTTCTGTCCCATGCCTACC | GQ340770 |
| *AtCHS* | AGAAGGGTTGGAGTGGGGT | CGTAGGTAGGTAGGCAGATAGA | AT5G13930 |
| *AtCHI* | CTTCGCTCTCTCCCCTACC | CCTTTTCGTCCTTGTTCTTCATC | AT3G55120 |
| *AtF3H* | CAGGGACGAAGATGAACGGC | AAGCAAAGAAGTCACGAGCG | AT3G51240 |
| *AtF3’H* | ATCCACCAACACCACTCTCG | GCTTCCTTTCACATCAACGCC | AT5G07990 |
| *AtDFR1* | CTTCGGGTTTCATCGGTTCAT | AGTAGCGTCTTGGCGTTTGG | AT5G42800 |
| *AtANS* | CGGTCCTCAAGTTCCCACAA | TCCCCAATCCAAAGATGCCT | AT4G22880 |
| *AtUFGT* | CGACGCAATCGCTATAAGG | ACTGCCTACTTATGTAATCGCAGA | AT5G54060 |
| *AtACT2* | CTTGCACCAAGCAGCATGAA | CTTTGCACGCAGTGTATGCTC | AT3G18780 |
