# Supplementary material for: An Anthocyanin-Related Glutathione S-Transferase, MrGST1, Plays an Essential Role in Fruit Coloration in Chinese Bayberry (Morella rubra)
Source: Front Plant Sci. 2022 Jun 8;13:903333. doi: 10.3389/fpls.2022.903333 (PMC9213753; doi:10.3389/fpls.2022.903333)
Supplement: Supplementary file 2 [file Table_2.DOCX]

**Table S2** Primers used for ectopic expression vector construction. The sequences of restriction sites are underlined.

| **Name** | **Forward/ Reverse primer Sequence (5′ to 3′)** | **Description** |
| --- | --- | --- |
| SK-MrGST1 F | AGGACAGCCCAAGCTGAGCTCATGGTTGTGAAAGTGTATGGTTCAA | Subclone to pGreen-SK Vector of MrGST1 |
| SK-MrGST1 R | TGATTTCAGCGAATTGGTACCCTAGTAACCAGCAAGCGTCAGTAAC | Subclone to pGreen-SK Vector of MrGST1 |
| SK-MrMYB1.1 F | CGCTCTAGAACTAGTGGATCCATGGAAGGCTCTTTAGGTGTACGA | Subclone to pGreen-SK Vector of MrMYB1.1 |
| SK-MrMYB1.1 R | CAGCGAATTGGTACCGGGCCCCTGGATCGAGAAAATCCCAAA | Subclone to pGreen-SK Vector of MrMYB1.1 |
| SK-MrbHLH1 F | CGCTCTAGAACTAGTGGATCCATGGCTGCACCGCCGAG | Subclone to pGreen-SK Vector of MrbHLH1 |
| SK-MrbHLH1 R | CAGCGAATTGGTACCGGGCCCCTACGAGTCATTGTGGGGTATAATTT | Subclone to pGreen-SK Vector of MrbHLH1 |
| LUC-MrGST1 F | CTATAGGGCGAATTGGGTACCGATTTTGATGACCGGCCAGA | Subclone to pGreen-LUC Vector of MrGST1 promoter |
| LUC-MrGST1 R | CGCTCTAGAACTAGTGGATCCCTTAATTCTATTGCCTAAAAAGCAGTTC | Subclone to pGreen-LUC Vector of MrGST1 promoter |
| LUC-MrGST1m1 F | TCGTGTTTCCCACTGACTTTGTCCAATGGACAA | Subclone to pGreen-LUC Vector of MrGST1m1 promoter |
| LUC-MrGST1m1 R | TTGTCCATTGGACAAAGTCAGTGGGAAACACGA | Subclone to pGreen-LUC Vector of MrGST1m1 promoter |
| LUC-MrGST1m2 F | TCTCTTTTTTTTTTTTTCGATCCAATATGCTAATT | Subclone to pGreen-LUC Vector of MrGST1m2 promoter |
| LUC-MrGST1m2 R | AATTAGCATATTGGATCGAAAAAAAAAAAAAGAGA | Subclone to pGreen-LUC Vector of MrGST1m2 promoter |
| LUC-MrGST1m3 F | AGGGTATGGGCCCCTGTGTCAAATCTAACTTTT | Subclone to pGreen-LUC Vector of MrGST1m3 promoter |
| LUC-MrGST1m3 R | AAAAGTTAGATTTGACACAGGGGCCCATACCCT | Subclone to pGreen-LUC Vector of MrGST1m3 promoter |
| LUC-MrGST1m4 F | TGGAAATATTTGGGGTGGACAAATTTCGCCCAG | Subclone to pGreen-LUC Vector of MrGST1m4 promoter |
| LUC-MrGST1m4 R | CTGGGCGAAATTTGTCCACCCCAAATATTTCCA | Subclone to pGreen-LUC Vector of MrGST1m4 promoter |
| LUC-MrGST1m5 F | ACTTCAAAGGTTGCTCCTTGGATCGAGCCGAGGCA | Subclone to pGreen-LUC Vector of MrGST1m5 promoter |
| LUC-MrGST1m5 R | TGCCTCGGCTCGATCCAAGGAGCAACCTTTGAAGT | Subclone to pGreen-LUC Vector of MrGST1m5 promoter |
| LUC-MrGST1m6 F | GTTTGGTTTCTGGAAGTCTTGATCGAAGAAGAAGA | Subclone to pGreen-LUC Vector of MrGST1m6 promoter |
| LUC-MrGST1m6 R | TCTTCTTCTTCGATCAAGACTTCCAGAAACCAAAC | Subclone to pGreen-LUC Vector of MrGST1m6 promoter |
| LUC-MrGST1m7 F | TTTCCACGTGACAACCCGCCAAAACTTAACGAG | Subclone to pGreen-LUC Vector of MrGST1m7 promoter |
| LUC-MrGST1m7 R | CTCGTTAAGTTTTGGCGGGTTGTCACGTGGAAA | Subclone to pGreen-LUC Vector of MrGST1m7 promoter |
| LUC-MrGST1m8 F | TCTGCTATATAAATAAGGGCAAGGAGCTTCGCA | Subclone to pGreen-LUC Vector of MrGST1m8 promoter |
| LUC-MrGST1m8 R | TGCGAAGCTCCTTGCCCTTATTTATATAGCAGA | Subclone to pGreen-LUC Vector of MrGST1m8 promoter |
| pAbAi-proMrGST1 F | GAAAAGCTTGAATTCGAGCTCTTTGGTGCATATAAGGTATTTTTCT | Subclone to pAbAi Vector of MrGST1 promoter |
| pAbAi-proMrGST1 R | ATACAGAGCACATGCCTCGAGCTTAATTCTATTGCCTAAAAAGCAG | Subclone to pAbAi Vector of MrGST1 promoter |
| pAbAi-pro3×MBS4 F | GAAAAGCTTGAATTCGAGCTCCCCTACCAACCAGCCTACCAACCAGCCTACCAACCAGCTCGAGGCATGTGCTCTGTAT | Subclone to pAbAi Vector of MBS4 motif in MrGST1 promoter |
| pAbAi-pro3×MBS4 R | ATACAGAGCACATGCCTCGAGCTGGTTGGTAGGCTGGTTGGTAGGCTGGTTGGTAGGGGAGCTCGAATTCAAGCTTTTC | Subclone to pAbAi Vector of MBS4 motif in MrGST1 promoter |
| pGADT7-MrMYB1.1 F | GCCATGGAGGCCAGTGAATTCATGGAAGGCTCTTTAGGTGTACGA | Subclone to pGADT7 Vector of MrMYB1.1 |
| pGADT7-MrMYB1.1 R | CCGCTGCAGGTCGACGGATCCTTATGGATCGAGAAAATCCCAAA | Subclone to pGADT7 Vector of MrMYB1.1 |
