# Supplementary material for: An Anthocyanin-Related Glutathione S-Transferase, MrGST1, Plays an Essential Role in Fruit Coloration in Chinese Bayberry (Morella rubra)
Source: Front Plant Sci. 2022 Jun 8;13:903333. doi: 10.3389/fpls.2022.903333 (PMC9213753; doi:10.3389/fpls.2022.903333)
Supplement: Supplementary file 3 [file Table_3.DOCX]

**Table S3** The linear-regression relationship between the expression level of anthocyanin-related genes in ‘Biqi’ Chinese bayberry fruit at six developmental stages.

|  | *MrCHS* | *MrCHI* | *MrF3H* | *MrF3'H* | *MrDFR1* | *MrDFR2* | *MrANS* | *MrUFGT* | *MrMYB1.1* | *MrbHLH1* | *MrWD40-1* |
| --- | --- | --- | --- | --- | --- | --- | --- | --- | --- | --- | --- |
| *MrCHI* | -0.297 |  |  |  |  |  |  |  |  |  |  |
| *MrF3H* | 0.672^**^ | -0.154 |  |  |  |  |  |  |  |  |  |
| *MrF3'H* | 0.831^***^ | -0.095 | 0.907^***^ |  |  |  |  |  |  |  |  |
| *MrDFR1* | 0.867^***^ | -0.147 | 0.698^**^ | 0.793^***^ |  |  |  |  |  |  |  |
| *MrDFR2* | 0.832^***^ | -0.515^*^ | 0.661^**^ | 0.672^**^ | 0.725^**^ |  |  |  |  |  |  |
| *MrANS* | 0.693^**^ | -0.254 | 0.717^**^ | 0.705^**^ | 0.729^**^ | 0.810^***^ |  |  |  |  |  |
| *MrUFGT* | 0.844^***^ | -0.163 | 0.721^**^ | 0.795^***^ | 0.866^***^ | 0.768^***^ | 0.674^**^ |  |  |  |  |
| *MrMYB1.1* | 0.852^***^ | -0.180 | 0.719^**^ | 0.853^***^ | 0.831^***^ | 0.729^**^ | 0.739^***^ | 0.807^***^ |  |  |  |
| *MrbHLH1* | -0.556^*^ | 0.359 | -0.394 | -0.423 | -0.428 | -0.689^**^ | -0.541^*^ | -0.462 | -0.466 |  |  |
| *MrWD40-1* | -0.373 | 0.906^***^ | -0.125 | -0.161 | -0.182 | -0.515^*^ | -0.207 | -0.176 | -0.234 | 0.324 |  |
| *MrGST1* | 0.649^**^ | -0.175 | 0.743^***^ | 0.660^**^ | 0.828^***^ | 0.760^***^ | 0.835^***^ | 0.785^***^ | 0.714^**^ | -0.410 | -0.088 |

* Significant difference at *P* < 0.05 level, ** Significant difference at *P* < 0.01 level, *** Significant difference at *P* < 0.001 level.
