# Supplementary material for: An Anthocyanin-Related Glutathione S-Transferase, MrGST1, Plays an Essential Role in Fruit Coloration in Chinese Bayberry (Morella rubra)
Source: Front Plant Sci. 2022 Jun 8;13:903333. doi: 10.3389/fpls.2022.903333 (PMC9213753; doi:10.3389/fpls.2022.903333)
Supplement: Supplementary file 4 [file Table_4.DOCX]

**Table S4** The linear-regression relationship between the expression level of anthocyanin-related genes in ripe fruit of 12 Chinese bayberry cultivars.

|  | *MrCHS* | *MrCHI* | *MrF3H* | *MrF3'H* | *MrDFR1* | *MrDFR2* | *MrANS* | *MrUFGT* | *MrMYB1.1* | *MrbHLH1* | *MrWD40-1* |
| --- | --- | --- | --- | --- | --- | --- | --- | --- | --- | --- | --- |
| *MrCHI* | 0.700* |  |  |  |  |  |  |  |  |  |  |
| *MrF3H* | 0.643* | 0.921*** |  |  |  |  |  |  |  |  |  |
| *MrF3'H* | 0.819** | 0.891*** | 0.920*** |  |  |  |  |  |  |  |  |
| *MrDFR1* | 0.913*** | 0.800** | 0.796** | 0.898*** |  |  |  |  |  |  |  |
| *MrDFR2* | 0.776** | 0.587* | 0.633* | 0.822** | 0.845** |  |  |  |  |  |  |
| *MrANS* | 0.273 | 0.796** | 0.730** | 0.548 | 0.402 | 0.087 |  |  |  |  |  |
| *MrUFGT* | 0.920*** | 0.817** | 0.817** | 0.929*** | 0.990*** | 0.883*** | 0.417 |  |  |  |  |
| *MrMYB1.1* | 0.122 | 0.218 | 0.206 | 0.138 | 0.209 | -0.160 | 0.291 | 0.128 |  |  |  |
| *MrbHLH1* | 0.312 | 0.504 | 0.521 | 0.457 | 0.438 | 0.025 | 0.488 | 0.367 | 0.732** |  |  |
| *MrWD40-1* | 0.774** | 0.404 | 0.328 | 0.541 | 0.586* | 0.371 | 0.238 | 0.584* | 0.304 | 0.418 |  |
| *MrGST1* | 0.597* | 0.810** | 0.694* | 0.799** | 0.720** | 0.589* | 0.621* | 0.729** | 0.100 | 0.500 | 0.494 |

* Significant difference at *P* < 0.05 level, ** Significant difference at *P* < 0.01 level, *** Significant difference at *P* < 0.001 level.
