# Supplementary material for: An Anthocyanin-Related Glutathione S-Transferase, MrGST1, Plays an Essential Role in Fruit Coloration in Chinese Bayberry (Morella rubra)
Source: Front Plant Sci. 2022 Jun 8;13:903333. doi: 10.3389/fpls.2022.903333 (PMC9213753; doi:10.3389/fpls.2022.903333)
Supplement: Supplementary file 5 [file Table_5.DOCX]

**Table S5** *Cis*-acting elements present in the promoter sequence of *MrGST1*.

|  | Motif | Strand | Position | Sequence | Function |
| --- | --- | --- | --- | --- | --- |
| Core promoter element | TATA-box | + | 126 | TATA | core promoter element around -30 of transcription start |
| Enhancer element | CAAT-box | + | 11 | CAAT | common cis-acting element in promoter and enhancer regions |
| Stress responsive element | GC-motif | + | 156 | CCCCCG | enhancer-like element involved in anoxic specific inducibility |
| Hormone responsive element | DPBFCOREDCDC3 | + | 2041 | ACACTTG | cis-acting element involved in the abscisic acid responsiveness |
|  |  | - | 1519 | ACACGAG |  |
|  | ACGTT-box | + | 967 | AACGTT |  |
|  | ABRE | + | 1023 | AACGTGG |  |
|  |  | - | 166 | CACGTGA |  |
|  | P-box | - | 918 | CCTTTTG | gibberellin-responsive element |
|  | GARE | - | 897 | TAACAGA |  |
|  | TATC-box | + | 605 | TATCCCA |  |
|  | AuxRE | + | 518, 2139 | TGTCTC | auxin-responsive element |
|  |  | - | 2046 | TGTCCCAT |  |
|  | TGA-element | + | 563 | AACGAC |  |
|  | AuxRR-core | + | 830 | GGTCCAT |  |
|  | NTBBF1 | - | 755, 772 | ACTTTA |  |
|  | ERE | + | 777, 1163 | ATTTTAAA | ethylene-responsive element |
|  |  | - | 1078, 1085 | ATTTTAAA |  |
| Temperature responsive element | LTRECOREATCOR15 | + | 384, 491 | CCGAC | Core of low temperature responsive element |

**Table S5 (Continued)**

|  | Motif | Strand | Position | Sequence | Function |
| --- | --- | --- | --- | --- | --- |
| Light responsive element | Gbox | + | 38 | TACGTG | cis-acting regulatory element involved in light responsiveness |
|  |  | + | 169, 330, 1026 | CACGTG |  |
|  |  | + | 977, 1910, 2208 | ACGTG |  |
|  | AT1-motif | - | 695 | AATTATTTTTTATT | part of a light responsive module |
|  | Box 4 | - | 1070, 1106 | ATTAAT | part of a conserved DNA module involved in light responsiveness |
|  |  | + | 1255, 1711, 1851 | ATTAAT |  |
|  | GT-1 | + | 68, 636 | GGAAAT | Consensus GT-1 binding site in many light-regulated genes |
|  |  | - | 56, 459, 874, 963, 1528, 1543 | GAAAAA |  |
|  |  | - | 170 | GGAAAT |  |
|  |  | - | 398, 1445 | GAAAAT |  |
|  |  | + | 734 | GATAAA |  |
|  |  | + | 766, 1134, 1180, 1472 | GAAAAA |  |
|  |  | + | 1633 | GAAAAT |  |
|  |  | - | 1739 | GATAAT |  |
|  |  | + | 1836 | GTAAAG |  |
|  | Ibox | + | 734, 1953 | GATAA | Conserved sequence upstream of light-regulated genes |
|  |  | - | 893, 1738 | GATAA |  |
| Transcription factor binding sites | Dof | + | 186, 358, 451, 578, 754, 915, 988, 1123, 1190, 1228, 1271, 1469, 1484, 1761, 1833, 1994 | AAAG |  |
|  |  | - | 20, 59, 179, 485, 513, 885, 953, 1433, 1440, 1654, 1775, 1964, 2119, 2175 | AAAG |  |

**Table S5 (Continued)**

|  | Motif | Strand | Position | Sequence | Function |
| --- | --- | --- | --- | --- | --- |
| Transcription factor | MYB | + | 116 | CGGTTG |  |
| binding sites |  | + | 153 | CCGTTA |  |
|  |  | - | 198 | GGTTGTTG |  |
|  |  | + | 348 | TACCAACC |  |
|  |  | + | 622 | CAGTTA |  |
|  |  | + | 897 | CTGTTA |  |
|  |  | - | 1413 | TACCAACC |  |
|  |  | - | 1497 | CAGTTA |  |
|  | MYC | + | 165, 326 | CACGTG |  |
|  |  | + | 265 | CATTTG |  |
|  |  | + | 1624, 2040 | CACTTG |  |
|  |  | + | 2021, 2053 | CAATTG |  |
|  |  | + | 2113 | CAAGTG |  |
|  |  | + | 2182 | CATCTG |  |
|  | RAV | + | 203, 268, 925 | CAACA |  |
|  | WRKY | - | 194, 201, 369, 919 | TGACT |  |
|  |  | + | 1505, 1768, 1857, 1916 | TGACT |  |
|  |  | + | 2220 | TGACC |  |
|  | ARR | - | 135 | AGATT |  |
|  |  | + | 172, 1121, 1303, 1318 | AGATT |  |
|  |  | - | 238, 1882 | TGATT |  |
|  |  | + | 409, 876 | CGATT |  |
|  |  | + | 740, 778 | GGATT |  |
