# Supplementary material for: An Anthocyanin-Related Glutathione S-Transferase, MrGST1, Plays an Essential Role in Fruit Coloration in Chinese Bayberry (Morella rubra)
Source: Front Plant Sci. 2022 Jun 8;13:903333. doi: 10.3389/fpls.2022.903333 (PMC9213753; doi:10.3389/fpls.2022.903333)
Supplement: Supplementary file 6 [file Table_6.DOCX]

**Table S6** Original and mutated sequences of eight MYB binding sites in *MrGST1* promoter sequence.

|  | Original sequence | Mutated sequence |
| --- | --- | --- |
| *MrGST1m1* | CGGTTG | GGCAAG |
| *MrGST1m2* | CCGTTA | GCCAAA |
| *MrGST1m3* | CAACAACC | CTTGATCG |
| *MrGST1m4* | TACCAACC | TTGGATCG |
| *MrGST1m5* | CAGTTA | GACAAA |
| *MrGST1m6* | CTGTTA | GTCAAA |
| *MrGST1m7* | GGTTGGTA | CGATCCAA |
| *MrGST1m8* | TAACTG | TTTGTC |
